# Exploring Biofilm-Related Traits and Bile Salt Efficacy as Anti-Biofilm Agents in MDR *Acinetobacter baumannii*

**DOI:** 10.3390/antibiotics13090880

**Published:** 2024-09-13

**Authors:** Verica Aleksic Sabo, Dušan Škorić, Suzana Jovanović-Šanta, Petar Knezevic

**Affiliations:** 1 Department of Biology and Ecology, Faculty of Sciences, University of Novi Sad, Trg Dositeja Obradovica 2, 21000 Novi Sad, Serbia; verica.aleksic@dbe.uns.ac.rs; 2 Department of Chemistry, Biochemistry and Environmental Protection, Faculty of Sciences, University of Novi Sad, Trg Dositeja Obradovica 3, 21000 Novi Sad, Serbia; dusan.skoric@dh.uns.ac.rs (D.Š.); suzana.jovanovic-santa@dh.uns.ac.rs (S.J.-Š.)

**Keywords:** biofilm, autoaggregation, *Acinetobacter baumannii*, Na-deoxycholate, Na-chenodeoxycholate

## Abstract

*Acinetobacter baumannii* has been designated as a critical priority pathogen by the World Health Organization for the development of novel antimicrobial agents. This study aimed to investigate both the phenotypic and genotypic traits of multidrug-resistant (MDR) *A. baumannii* strains, along with the effects of natural bile salts on biofilm formation. The research analyzed phenotypic traits, including autoaggregation, hydrophobicity, twitching motility, lectin production, and biofilm formation, as well as genotypic traits such as the presence of *bap* and *bla*_PER-1_ genes in twenty wound and eight environmental MDR *A. baumannii* isolates. While all strains were identified as good biofilm producers, no statistically significant correlation was detected between the examined traits and biofilm formation. However, differences in biofilm production were observed between environmental and wound isolates. The natural bile salts Na-cholate, Na-deoxycholate, and Na-chenodeoxycholate demonstrated effective anti-*A. baumannii* activity (MIC = 0.25–10 mg mL^−1^), with significant anti-biofilm effects. Na-deoxycholate and Na-chenodeoxycholate inhibited 94–100% of biofilm formation at super-MIC concentrations (8–32 mg mL^−1^). This study underscores the urgent need for innovative strategies to combat antibiotic resistance and biofilm formation in *A. baumannii*, highlighting the potential of natural bile salts as promising biofilm inhibitors and encouraging further research into their modification and combination with other antimicrobials.

## 1. Introduction

*Acinetobacter baumannii* is a clinically dominant species with a pronounced tendency to induce nosocomial infections, especially in intensive care units [1,2]. The incidence of hospital infections caused by this bacterium in surgical departments and in intensive care units is on the rise. As of 2018, *A. baumannii* infections accounted for approximately 2% of all healthcare-associated infections in the United States and Europe, with rates nearly double in Asia and the Middle East [3]. *A. baumannii* represents a significant opportunistic pathogen, as it rapidly develops resistance to a large number of antibiotics, including most β-lactam antibiotics and aminoglycosides [4]. Although other Gram-negative pathogens may exhibit higher infection rates, about 45% of all *A. baumannii* isolates are classified as multidrug-resistant. This is a four-fold higher rate than in other Gram-negative pathogens (e.g., *Pseudomonas aeruginosa* and *Klebsiella pneumoniae*), and the rates can be even up to 70% in Latin America and the Middle East [3].

One of the main reasons for the limited number of currently available antimicrobial agents effective in eradicating *A. baumannii* infection is that most antibiotics affect planktonic bacterial cells. However, bacteria in its natural environment most commonly live in form of biofilm, which additionally provide phenotypic resistance to conventional antibiotics. It has been reported that 65% of human infections are related to biofilm, which show up to 10- to 1000-fold higher resistance compared to their planktonic cells [5,6]. The formation of biofilms, among other numerous benefits, also allows greater gene transfer between bacteria, including antibiotic resistance genes, so that bacteria in biofilm are more resistant to antimicrobial agents and are considered multidrug (MDR), extensively drug (XDR), or even pandrug (PDR) resistant. It is precisely the potential of the genomic species *A. baumannii* to form a biofilm that can explain its extraordinary antibiotic resistance [5,6,7]. The potential of *A. baumannii* to form biofilm contributes to its survival in adverse environmental conditions, including hospital environments and medical device surfaces. This bacterium possesses the ability to form biofilm on biotic surfaces, such as wounds and lung tissue of patients with cystic fibrosis, as well as on abiotic surfaces, primarily on medical devices and equipment, such as catheters, endotracheal tubes, and other objects [8]. Thus, it is becoming evident that biofilm-forming ability can be considered one of the main virulence factors common to a large number of *A. baumannii* clinical isolates [9,10,11,12].

The annual incidence of *A.baumannii* resistance rate creates challenges in the clinic [13]. According to the American Centers for Disease Control and Prevention (CDC), carbapenem-resistant *A. baumannii* is responsible for more than 700 deaths, 8500 infections, and $281 million in healthcare costs annually in the USA with limited treatment options. *A. baumannii* is being recognized as a source of global outbreaks and epidemics by the CDC, especially due to its increasing resistance to commercially available antibiotics [14]. The World Health Organization (WHO) has ranked carbapenem-resistant *A. baumannii* as a critical priority pathogen on its global priority list of antibiotic-resistant bacteria for development and/or finding novel antimicrobial agents [13].

Due to a lack of efficient antimicrobials, recently bile salts are increasingly attracting the attention of the researchers and scientific community in general [15,16,17]. Traditional medicinal use of bile salts dates back to ancient times. The first records found date from the Graeco-Roman period, the Anglo-Saxon period, and also from the period of the Zhou dynasty (c. 1046–256 BCE) [16,17]. Bile salts are biomaterials reminiscent of traditional surfactants with peculiar structure and self-assembled topologies [15]. They are potent antimicrobial agents, representing an important component of innate defenses in the intestine and giving protection against invasive organisms. Furthermore, they play an important role in determining the microbial ecology of the intestine, and alterations in their levels can lead to increased colonization by pathogens. The amphipathic nature of these steroid molecules, originating from and associated with the specific conformation of the rings in the steroid nucleus, makes bile salts potent antibacterial agents mainly due to their membrane-damaging effects [18]. This is proven by the synergistic effect of bile salts and antibiotics with intracellular mechanisms of action [16]. Bile salts also inhibit the bioenergetics processes of microorganisms in vitro by intracellular acidification, dissipation of the proton motive force, and induction of DNA damage and protein denaturation [19,20,21]. The aim of this study was to examine phenotypic and genotypic traits in relation to biofilm, as well as the influence of natural bile salts on the biofilm formation process in MDR *A. baumannii* strains.

## 2. Results

### 2.1. Biofilm-Related A. baumannii Phenotype Characteristics

*A. baumannii* strains possessed most of the investigated phenotypic properties (autoaggregation, hydrophobicity, swarming motility, and lectin production) important in the biofilm formation process. Also, all strains possessed the ability to form a biofilm in vitro on polystyrene surfaces.

**Figure 1:**
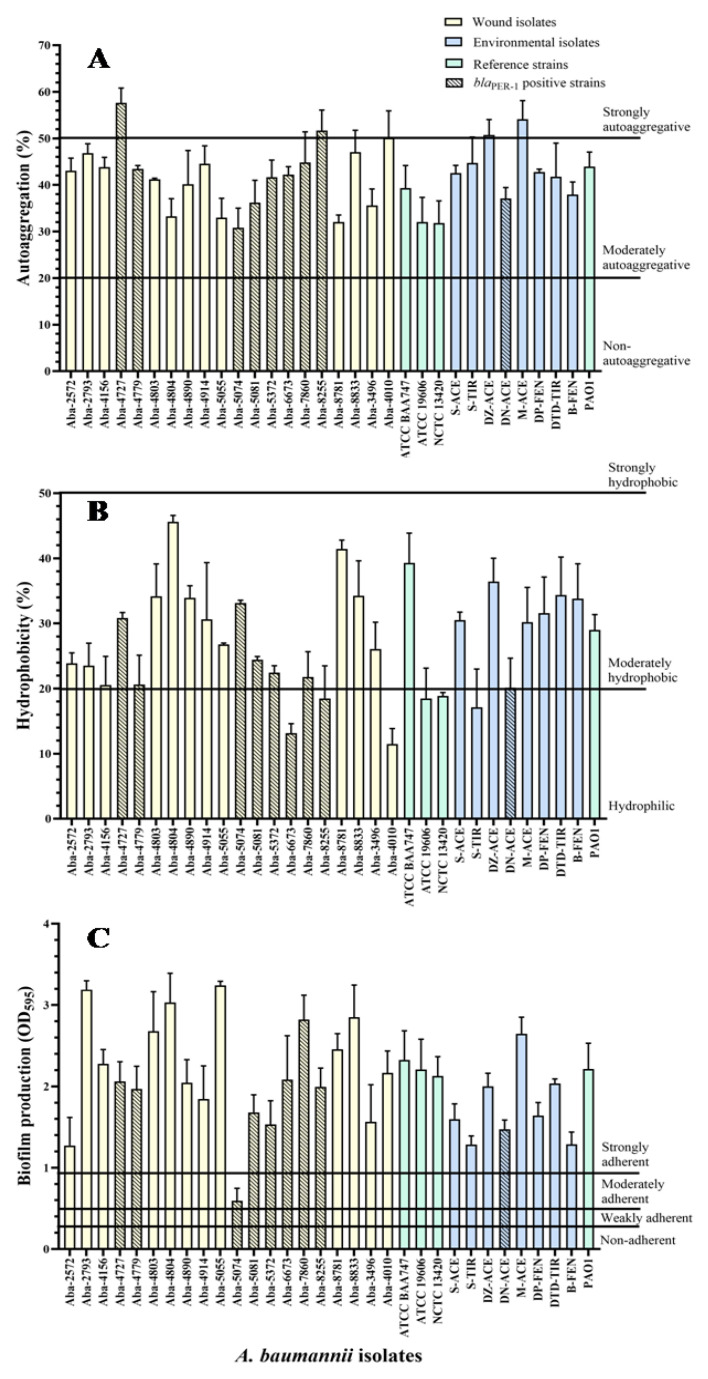
Autoaggregation (**A**), hydrophobicity (**B**), and biofilm production (**C**) of the MDR *A. baumannii* isolates.

The results of the *A. baumannii* wound and environmental isolates autoaggregation degree, as well as two strains of the members of the *A. calcocaceticus*–*A. baumannii* (Acb) complex, are shown in Figure 1A. Most of the wound isolates showed a moderate level of cells autoaggregation, ranging from 30.8 to 50%, except for three MDR strains, which proved to be extremely autoaggregative (Aba-4727, Aba-8255, and Aba-4010). Similarly, environmental isolates generally expressed a moderate degree of autoaggregation, ranging from 37% to 44.7%, except for two highly autoaggregative strains, Aba-DZ-Ace (50.7%) and Aba-M-Ace (54.1%). The strain from the wound that proved to be the most autoaggregative in comparison to all tested *A. baumannii* isolates was Aba-4727 (57.6%), while the strain Aba-5074 (30.8%), also originating from the wound, proved to be the least autoaggregative, when all investigated strains were taken into account. There was no statistically significant difference in the degree of autoaggregation between isolates originating from human pathological material and isolates originating from the environment (*p* = 0.78).

The results of the degree of *A. baumannii* cell hydrophobicity, as well as two strains of members of the Acb complex, are shown in Figure 1B. The largest number of wound isolates showed a moderate degree of hydrophobicity, ranging from 20.5% to 45.6%, with the exception of five isolates that were hydrophilic. The wound isolate Aba-4804 proved to be the most hydrophobic. All the environmental isolates expressed a moderate degree of hydrophobicity ranging from 20% to 36.4%, except the hydrophilic strain Aba-S-Tyr. The Aba-5074 wound isolate proved to be the least hydrophobic (7.1%) when all the examined strains were taken into account. There was no statistically significant difference in the degree of hydrophobicity between the isolates of different origins (i.e., human pathological material and environmental) (*p* = 0.89). Statistically significant correlation between hydrophobic and autoaggregation properties was not determined (τ = −0.01; *p* = 0.93).

The results of the bacterial twitching motility, as one of the important characteristics for determining the potential of biofilm formation, are shown in Table 1. The ability of a twitching movement, i.e., motility using type IV pili, was observed in all tested isolates. The size of the diffuse blur zone around the inoculation site after 48 h for the wound isolates ranged from 9.5 mm to 51.5 mm, while for the environmental strains it was from 12.0 mm to 52.5 mm. All isolates were classified as motile: 43.75% demonstrated intermediate motility, while 56.25% exhibited high motility. There was no statistically significant difference in the degree of twitching motility between the isolates originating from the wounds and the isolates originating from the environment (*p* = 0.059).

Statistically significant linear correlation was not found between the twitching motility and the autoaggregation (τ = −0.03; *p* = 0.82), nor the hydrophobicity of the cells (τ = 0.10; *p* = 0.44).

The results of *A. baumannii* lectin haemagglutination for the erythrocytes of various blood types are shown in Table 1. It can be clearly observed that lectins produced by *A. baumannii* isolates of different origins, as well as two isolates of the Acb complex, exhibited haemagglutinase activity in all tested blood types.

The results of semi-quantification of the produced lectins by *A. baumannii* isolates and two isolates of the Acb complex are also shown in Table 1. The titre of haemagglutination ranged from 1:4 to 1:64. The lectins titres (i.e., double dilutions) that have led to haemagglutination in most strains were 1:16 and 1:32.

The lectin titre of reference strains was slightly lower than for the other isolates. The titre of haemagglutination of the isolates originating from the human pathological material was slightly higher than the titre of the environmental strains. Wound isolates Aba-4804 and Aba-4890 produced most of the lectins, with the titre of haemagglutination 1:64. However, on the basis of the obtained results, there was no statistically significant difference between the degree of lectin production for strains originating from human pathological material in relation to environmental isolates (*p* = 0.87). Also, there was no statistically significant correlation between the degree of production of lectins and other examined properties of tested strains–autoaggregation (τ = −0.05, *p* = 0.68), hydrophobicity (τ = 0.02, *p* = 0.86) and twitching motility (τ = −0.03, *p* = 0.81).

The degree of biofilm production for wound and environmental isolates on polystyrene is shown in Figure 1C. All the examined isolates proved to be strongly adherent, except the Aba-5074 wound isolate, which proved to be moderately adherent. It is noticeable that the degree of biofilm production in wound isolates is highest for the Aba-5055 strain, while the in environmental isolates is greatest in the Aba-M-Ace strain. Statistical analysis of the results showed a statistically significant difference in the biofilm production rate of the strains of different origin (*p* = 0.036).

There was no statistically significant linear correlation between the biofilm production degree and the level of lectin production (τ = 0.09, *p* = 0.47), as well as other tested properties, no statistically significant linear correlation was established. Using the multiple-regression statistical test, the multiple correlation coefficient (R = 0.387, *p* = 0.358, meaning *p* > 0.05) was determined, indicating the low quality of predicting using biofilm production degree as a dependent variable based on the tested properties of autoaggregation, hydrophobicity, twitching motility, and the production of lectins. The determined significance of these properties was in the range 0.061–0.924, whereby, as a slightly more important feature, compared with others, it distinguished the cell’s hydrophobicity property (*p* = 0.061).

### 2.2. Biofilm-Related A. baumannii Genotype Characteristics

The presence of the *bap* (biofilm-associated protein) gene encoding the Bap protein, important for the biofilm formation process, was determined by analyzing the genomic DNA *A. baumannii* isolates and two Acb complex isolates in all of the tested isolates (Table 1).

The presence of *bla*_PER-1_ gene was detected in a total of nine isolates (29.0%), eight wound isolates (Aba-4727, Aba-4779, Aba-5074, Aba-5081, Aba-5372, Aba-6673, Aba-7860, and Aba-8255), and one environmental isolate (Aba-DN-Ace) (Table 1). The negative statistically significant linear correlation was confirmed for the production of the PER-1 (*Pseudomonas* extended resistance) type β-lactamase and the potential of the cells to form a biofilm (τ = −0.27, *p* = 0.033), as well as the cell hydrophobicity (τ = −0.41, *p* = 0.001). In contrast to these properties, the analysis of the correlation between the production of PER-1 type β-lactamase and the autoaggregation (τ = 0.05, *p* = 0.68) and twitching motility (τ = 0.11, *p* = 0.39) a statistically significant linear correlation was not detected.

### 2.3. Effect of Bile Salts on MDR A. baumannii

The results of the natural bile salts anti-*A. baumannii* activity are shown in Table 2. Determined minimal inhibitory concentration (MIC) values ranged from 0.25 to 10.1 mg mL^−1^. The best antibacterial effect expressed Na-deoxycholate with MIC values 0.25–0.8 mg mL^−1^ against MDR isolates. Good activity expressed bile salt Na-chenodeoxycholate, with MICs 0.5–2 mg mL^−1^. Na-cholate had the weakest activity on MDR *A. baumannii*; it inhibited bacterial growth in higher concentrations (MICs were 0.7–10.1 mg mL^−1^) compared to other used natural bile salts.

**Figure 2:**
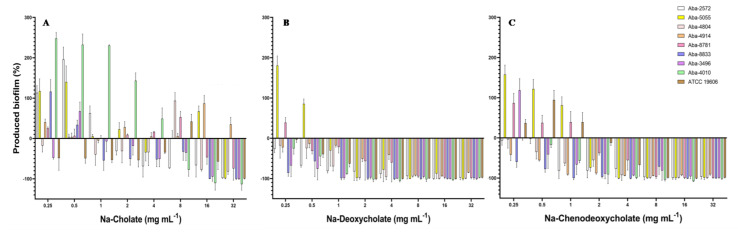
Effect of the natural bile salts on the biofilm-forming ability of *A. baumannii* (**A**) Na-Cholate, (**B**) Na-Deoxycholate, (**C**) Na-Chenodeoxycholate.

Biofilm inhibitory concentrations required to inhibit biofilm formation for 50% (BIC_50_) and 90% (BIC_90_) are shown in Table 2. Two bile salts (Na-DCA and Na-CDCA) expressed good anti-biofilm activity with BIC_50_ in range 0.25–4 mg mL^−1^ and BIC_90_ in range 1–8 mg mL^−1^. The effect of the natural bile salts on the process of biofilm formation is shown in Figure 2. All tested bile salts showed an inhibitory effect on the process of biofilm formation of MDR *A. baumannii* isolates, reducing the degree of biofilm produced. Na-deoxycholate proved to be the most effective, but the remaining two bile salts also inhibited the process of biofilm formation in MIC concentrations, while lower, i.e., sub-inhibitory concentrations of these substances in some strains slightly stimulated biofilm production (Figure 2).

When Na-cholate was applied as an anti-biofilm agent, the most efficient concentrations were 16 and 32 mg mL^−1^, which inhibited biofilm production for 50–100%, depending on the bacterial strain. Only for the strain Aba-4914 (previously characterized as the most resistant strain in the collection) the inhibition of biofilm formation was not achieved. On the contrary, Na-deoxycholate inhibited biofilm in all tested strains when MIC and super-MIC concentrations were applied. The higher concentrations of this bile salt (8–32 mg mL^−1^) almost completely inhibited biofilm formation (94–100%) in most of the strains. In concentrations 1–4 mg mL^−1^ Na-DCA inhibited biofilm for 20–100%, while in concentrations 0.25 and 0.5 mg mL^−1^ inhibition was 5–75%, depending on the strain (Figure 2B). Na-chenodeoxycholate expressed also good inhibitory activity. In concentrations 8–32 mg mL^−1^ inhibition was 80–100%, while with concentrations 2 and 4 mg mL^−1^ inhibition was 12–100% (Figure 2C). In sub-inhibitory concentrations 0.25–1 mg mL^−1^ biofilm reduction ranged 2.5–100%, while stimulation of biofilm production in sub-inhibitory concentrations was detected in some strains (Aba-4914, Aba-5055, Aba-8781, and ATCC 19606) (Figure 2).

**Figure 3:**
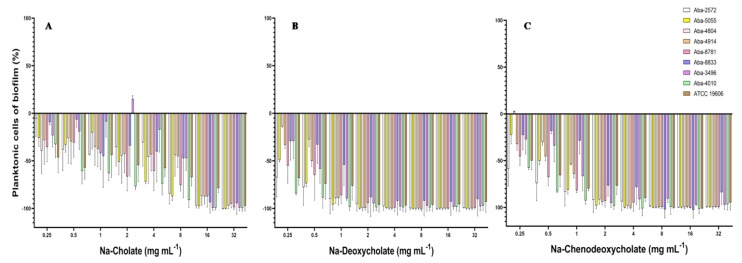
Effect of the natural bile salts on *A. baumannii* planktonic cells during biofilm formation (**A**) Na-Cholate, (**B**) Na-Deoxycholate, (**C**) Na-Chenodeoxycholate.

The effect on the inhibition of the planktonic cells present in the medium during the biofilm formation process was significant. All three tested natural bile salts in concentrations of 16 and 32 mg mL^−1^ inhibited planktonic cells for more than 90% (Figure 3). At concentrations of 8 and 4 mg mL^−1^ of Na-deoxycholate or Na-chenodeoxycholate planktonic cells inhibition was also above 90% (Figure 3B,C). In lower concentrations (0.25–1 mg mL^−1^) these two bile salts inhibited these planktonic cells for 15 to 98%. Na-cholate in these sub-inhibitory concentrations inhibited planktonic cells during biofilm formation for 5 to 60% (Figure 3A).

## 3. Discussion

The *A. baumannii* ability to colonize both biotic and abiotic surfaces and to grow in a form of biofilm, together with its MDR phenotype, seems to play an important role in the remarkable capacity of the microorganism to persist and spread, especially in the hospital environment [12,22].

Two processes, co-aggregation and auto-aggregation, are responsible for the initial phases of biofilm formation [23]. In our study, most *A. baumannii* isolates (83.9%) exhibited moderate auto-aggregation, while 16.1% were highly auto-aggregative. Previous research indicated that *A. baumannii* strains from wound infections and bacteremia had lower aggregative properties compared to respiratory and environmental strains [24], but this difference was not observed in isolates from Serbia. This suggests that the spread of the favorable traits, i.e., genes, between strains of different origins, is even more common than it is suspected. The phenomena of auto-aggregation and co-aggregation of bacteria are considered important processes in the formation of coherent structures such as biofilms. However, no significant correlation was found between auto-aggregation and biofilm production in wounds (τ = −0.10; *p* = 0.51) or environmental isolates (τ = 0.29; *p* = 0.32).

The study further revealed that 80.7% of the *A. baumannii* strains were moderately hydrophobic, while 19.3% were hydrophilic. A hydrophobic cell surface may provide an advantage in vivo by increasing resistance to phagocytosis and promoting tissue colonization in wounds. However, cell surface hydrophobicity showed no significant correlation with cell adhesion for both wound and environmental *A. baumannii* isolates (τ = 0.20; *p* = 0.11). Similarly, little to no influence of hydrophobicity on adhesion has been reported for the Gram-positive bacterium *Staphylococcus epidermidis* [25]. Although most of the biofilm-forming *A. baumannii* strains exhibit moderate hydrophobicity, this trait is not essential for biofilm development but rather has a supporting role. This indicates that cellular hydrophobicity alone is not a reliable indicator of biofilm formation potential in *A. baumannii*.

It is well known that *A. baumannii* has the ability to rapidly spread across both biotic and abiotic surfaces. Although it lacks flagella and swimming motility, this rapid spread is attributed to twitching motility mediated by type IV pili [26]. This study assessed hydrophobicity, autoaggregation, and twitching motility to determine the strains’ ability to colonize wound surfaces and form biofilms. All tested *A. baumannii* strains displayed twitching motility on the contact surface between the glass and medium containing 1.5% agar. While no statistically significant difference in motility was observed between wound and environmental isolates (*p* = 0.059), 65% (13/20) of wound isolates were highly motile, compared to 25% (2/8) of environmental strains. According to the available literature, pili on *A. baumannii* cells enhance adherence to epithelial cells, which is crucial for initial colonization and infection establishment [27]. Production of pili type IV is essential for biofilm formation, particularly on medical surfaces such as polystyrene [28]. Despite this, in vitro tests showed no significant correlation between twitching motility and biofilm formation on polystyrene (τ = 0.11; *p* = 0.37), nor between twitching motility and other cell traits. Similarly, McQueary and Actis [29] reported that there were significant variations among the properties of all tested *A. baumannii* strains (ATCC 19606 and 8 clinical isolates), which did not result in direct correlations between biofilm phenotype and examined cell traits that could affect the process of biofilm formation on abiotic surfaces (hydrophobicity, twitching motility, presence of *CsuA/B* gene). Considering the findings and the fact that all the examined wound and environmental strains exhibited twitching motility, the presence of type IV pili on the cell surface, together with other traits, plays a role in the formation of *A. baumannii* biofilm.

Another feature examined in *A. baumannii* was its ability to produce lectins. These proteins are ubiquitous in nature and can be found in other organisms [30]. Numerous bacterial strains have the ability to produce surface lectins, most often in the form of fimbria, which are short and numerous filamentous assemblages of protein subunits. The original name for lectin was hemagglutinin or agglutinin, due to its ability to agglutinate erythrocytes. Lectins, i.e., hemagglutinins are a heterogeneous group of proteins or glycoproteins that possess at least one non-catalytic domain that is reversibly bound to specific monosaccharides or oligosaccharides. The primary function of bacterial lectins is to facilitate the interconnection between bacterial and host cells during the initial phases of infection [31]. To further investigate *A. baumannii*, this study not only assessed its ability to produce lectins but also conducted a semi-quantitative analysis of their production and examined their specificity to different human blood types. All strains were found to produce extracellular lectins after 24 h of growth at 37 °C in a liquid nutrient medium, with *A. baumannii* lectins showing hemagglutination reactivity specific to O Rh+, A Rh+, and B Rh+ blood types. The hemagglutination titers for wound isolates were similar to those of environmental strains, ranging from 1:4 to 1:64. These findings are consistent with previous reports [32,33], which confirmed the hemagglutination ability of *A. baumannii* isolates from patients with respiratory tract infections, with titers ranging from 1:8 to 1:128 [33]. Some studies have shown that the formation of biofilm, gelatinous activity, and the ability of hemagglutination in *A. baumannii* strains are in relation to their pathogenicity [34]. Lectins as essential factors in the process of biofilm formation and bacterial cell binding to the host tissue can represent target molecules in the inhibition and prevention of infections and the formation of biofilms, especially on wounds. However, by comparing the degree of biofilm production, autoaggregation, and twitching motility with the established degree of lectin production, no statistically significant linear correlation between these properties of the tested *A. baumannii* isolates was detected. It is important to note that all strains exhibited biofilm production, as well as lectin production, autoaggregation, and twitching motility. The lack of a significant correlation between lectin production and other properties critical for bacterial adhesion and biofilm formation may be due to the greater relevance of lectins in in vivo adhesion compared to the in vitro conditions typically used to study adhesion.

*A. baumannii* possesses the ability to form biofilm on various surfaces such as glass, polycarbonate, polypropylene, and urinary catheters of silicone or latex [35]. The results obtained here confirm that *A. baumannii* isolates possess the ability of adhesion and biofilm formation on the polystyrene surface. The tested *A. baumannii* isolates exhibited a high degree of adhesion to polystyrene and were characterized as very adherent, similarly to other literature reports [29,36,37,38]. It is important to note that some of these materials are widely used in the production of medical equipment, and the established ability of *A. baumannii* to survive and persist on abiotic materials is associated with the spread of nosocomial infections. Additionally, the obligate aerobic nature of this pathogen promotes the formation of thick cell conglomerates in the air–liquid phase contact zone [39], which may occur in endotracheal tubes, urinary, and venous catheters. It has been reported that the cells of the *A. baumannii* reference strain ATCC 19606 form denser aggregates at the contact of the air–liquid phase on the walls and bottom of the plates and tubes [7]. This emphasizes that the biofilm of the reference strain in liquid cultures grows towards the surface, i.e., in the direction of contacting the air–liquid phase along the walls of the plate, a pattern also observed in the isolates tested here when staining biofilm with crystal violet. This phenomenon is not caused by mixing and moving the liquid substrate, as the microtiter plates were incubated under stationary conditions, so it can be assumed that it is a consequence of the phenotypic traits of this pathogen (aerobic, oxidative type of metabolism, hydrophobicity, adhesion, etc.). Our findings indicate that environmental isolates exhibit lower biofilm production compared to wound isolates. Statistical analysis confirmed this difference to be significant (*p* = 0.036). These results suggest that clinical isolates retain properties favoring biofilm growth, despite the absence of a correlation between examined phenotypic characteristics and biofilm production levels. Given that *A. baumannii* hospital and outpatient isolates from wounds were used, the observed difference in biofilm production highlights their strong adhesion and colonization abilities on wound surfaces. This finding explains why *A. baumannii* wound infections are often chronic and recurrent and underscores the importance of treating such infections as biofilm-associated.

The selected genotypic properties of the isolates were determined by detecting the presence of the genes for Bap protein and PER-1 type β-lactamase.

The bacterial adhesin Bap, present on the cell surface and conserved across different clinical isolates, was initially identified in *A. baumannii* strain 307-0294 by Loehfelm et al. [40]. It was postulated to participate in intercellular adhesion, binding with receptors on other bacterial cells and facilitating aggregation. It has been found that intercellular adhesion allows the maturation of biofilm after initial binding to the abiotic surface, but also that the production of Bap surface adhesion protein and pili plays a role in the initiation of biofilm formation [41,42]. Analyzing genomic DNA here tested *A. baumannii* isolates, in all tested strains, the presence of *bap* gene encoding Bap protein was detected. All the analyzed *A. baumannii* isolates have substantially formed a biofilm and possessed a *bap* gene, which agrees with earlier claims that this protein has multiple significant roles in the biofilm formation process, such as initial adhesion to abiotic surfaces, colonization of hosts, and stabilization of mature biofilm on the glass, affecting the thickness and biovolume of the biofilm. All mentioned points to the fact that Bap protein in the genomic species *A. baumannii* represents a very important factor in the biofilm formation process, but to date there are no studies describing potential environmental factors and conditions that would allow control of the differential expression of *bap* genes.

PER-1 type β-lactamase was first detected in France in 1991 with *P. aeruginos* strain isolated from a patient transported from Turkey and later detected in *Salmonella* spp. and *Acinetobacter* spp. in Turkey [42]. The strains of the genus *Acinetobacter* are ubiquitous in the hospital environment and therefore have frequent contact with ESBL-coding genes, but these plasmid-encoded enzymes were detected in *A. baumannii* for the first time by Sechi et al. [43]. PER-type enzymes provide a high degree of resistance to ceftazidime but lower levels of resistance to other cephalosporins and penicillins, while they have no effect against cefamycin and carbapenems. According to the obtained results for *A. baumannii* isolates, the presence of the *bla*_PER-1_ gene was detected in 31% of the total isolates, that is, in 40% of *A. baumannii* wound isolates. This is in correspondence to a previous report where the presence of PER-1 type β-lactamase in *Acinetobacter* strains was detected in 5 out of 8 hospitals, or 45.8% of strains (33 of 72) [43]. All the analyzed *A. baumannii* isolates were resistant to ceftriaxone with MICs in the range from 16 to >256 μg mL^−1^ [44], whereby the MIC value for all wound isolates carrying the *bla*_PER-1_ gene was >256 μg mL^−1^. Furthermore, there are several previous studies which indicate a possible relationship between the genes of PER-1 type β-lactamase and cell adhesion properties in strains of *A. baumannii* [11,27,43]. Accordingly, all eight wound isolates possessing the *bla*_PER-1_ gene proved to be very adherent and formed a substantial biofilm, with the exception of the Aba-5074 strain which proved to be moderately adherent and formed a lesser amount of biofilm. In addition, there is a negative statistically significant linear correlation between the presence of genes for the PER-1 type of β-lactamase and the potential of the cells to form a biofilm (τ = −0.27, *p* = 0.033), as well as the cell hydrophobic properties (τ = 0.41, *p* = 0.001). The negative correlation detected here corresponds to the results of several previous studies conducted in India, Turkey, and Spain [11,43,45]. However, there are also opposite reports, claiming that the adhesion of *A. baumannii* strains to the plastic surfaces was enhanced by the presence and expression of the *bla*_PER-1_ gene, although the mechanism mediated in this process remains unspecified [27]. The available literature data, together with the results obtained here, question the true relevance of the expression of the *bla*_PER-1_ gene in the formation of biofilm, i.e., the assumption of the existence of a relation between the *bla*_PER-1_ gene and different virulence determinants, in particular those involved in cell adhesion. This variability in the adherence of PER-1 producers, nevertheless, can be explained by studies showing that *bla*_PER-1_ disseminates on different gene fragments [42,43]. Therefore, the existence of virulence genes in some of the different gene packages is highly probable. Additionally, the differences in bacterial cell adhesion for abiotic and biotic surfaces may provide an explanation for the observed negative correlation in tests using in vitro abiotic surfaces, such as polistirene.

Despite identifying all MDR *Acinetobacter baumannii* strains as good biofilm producers, the study did not establish a clear connection between specific traits (like autoaggregation, hydrophobicity, twitching motility, lectin production, and the presence of the *bap* and *bla_PER-1_* genes) and the extent of biofilm formation. This lack of correlation may limit the ability to predict biofilm formation based on these traits alone, suggesting that other factors not examined in this study might be influencing biofilm production.

These numerous traits favor the resistance and biofilm production of the *A. baumannii* strains, raising the urgency for finding efficient solutions and/or alternatives for the antibiotics. Nature is an inexhaustible source of diverse molecules possessing potential for combating bacterial resistance. Throughout the course of infection, many pathogens encounter bactericidal conditions that threaten the viability of the bacteria and impede the establishment of infection. Bile is one of the most innately bactericidal substances present in humans, functioning to facilitate digestion while also aiding in reducing the bacterial burden in the gastrointestinal tract. The detected effect of natural bile salts on inhibition of MDR *A. baumannii* biofilm was notable, especially for Na-deoxycholate and Na-chenodeoxycholate. Detected BIC_50_ (0.25–4 mg mL^−1^) and BIC_90_ (1–8 mg mL^−1^) values were 1 × MIC to 8 × MIC (Table 2). These natural bile salts significantly eradicated 24 h pre-formed biofilms of MDR *A. baumannii* in a dose-dependent manner. Considering the fact that there are currently no clinically approved small-molecule biofilm inhibitors on the market in the United States and Europe, most recent studies have focused on testing of FDA-approved “non-antibiotic” drugs as potential anti-biofilm agents [46] and/or conducting virtual (in silico) screening of FDA-approved drugs for their anti-biofilm potential [47]. Comprehensive analyses of the bile response in *Acinetobacter baumannii* have been limited. To our knowledge, this is the first report confirming anti-biofilm activity of natural bile salts against MDR *A. baumannii*. One study describes the identification of taurine-conjugated bile acids as inhibitors of biofilm formation against both *Vibrio cholerae* and *Pseudomonas aeruginosa* [48]. However, according to other findings, 0.8% of Bacto bile salts promote *E. coli* O157:H7 growth under iron limiting conditions, suggesting that this strain uses bile salts as an environmental signal to adapt to changing conditions associated with the environment, such as the small intestine, including adaptation to an iron-scarce environment [49]. A recent study analyzed *A. baumannii* bile salt response in the context of activation of the bacterial quorum sensing system and modulation of virulence factors such as surface motility, biofilm, and type VI secretion system. The bile salts (Na-cholate 50% and Na-deoxycholate 50%, Sigma Aldrich, St. Louis, MO, USA) are usually used in in vitro tests at a concentration of 0.5%, as the physiological concentration in the human intestine ranges between 0.1 and 1.3% [50,51]. This correlates with the obtained Na-deoxycholate and Na-chenodeoxycholate anti-biofilm activity achieved in super-MIC concentrations (≥8 mg mL^−1^), expressing a pronounced effect on the process of biofilm formation, which was even greater on planktonic cells present in the medium during this process. This is promising, considering the fact that these planktonic cells enable further spread and dissemination of the biofilm.

As amphipathic molecules, bile salts disrupt the bacterial cell membrane, leading to cell death or leakage of intracellular components [52]. Detected high motility of *A. baumannii* strains enhanced their exposure to bile salts, potentially increasing their susceptibility to membrane disruption in planktonic cells. Also, bile salts can interfere with the adhesion of bacteria to surfaces, a critical initial step in biofilm formation [53]. It has been demonstrated that Na-cholate, Na-deoxycholate, and Na-chenodeoxycholate can modulate the adhesion and invasion of epithelial cells in several bacterial gut pathogens, including *Vibrio parahaemolyticus*, *Salmonella enterica* serovar Typhimurium, *Campylobacter jejuni*, *Escherichia coli*, *Bacteroides fragilis*, and *Shigella flexneri* [51,54,55,56,57,58]. It is postulated that bile and bile salts present in the small intestine may act as a signal for bacteria to initiate colonization of the epithelium [59,60]. However, this hypothesis is questionable. Unconjugated and conjugated bile salts at sub-inhibitory concentrations have been shown to inhibit *S. aureus* growth by disrupting the proton motive force and increasing membrane permeability [52], while *Salmonella enterica* serovar Typhimurium invasion is repressed in the presence of bile [55]. Additionally, bile salts (Na-cholate, Na-deoxycholate, and Na-chenodeoxycholate) exhibit an inhibitory effect on the biofilm formation of MDR *A. baumannii* wound isolates. These findings suggest that this phenomenon is selective for specific bile salt-pathogen combinations, indicating that bile salts may still play a significant role in controlling wound infections caused by biofilm-forming pathogens.

The inhibition of the initial phases of biofilm establishment on polistirene surface is of great importance for the potential treatment of the medical devices affected with biofilm of MDR opportunistic pathogens such as *A. baumannii* and *P. aeruginosa*, designated as the most common causative agents of problematic biofilm infections [61]. Furthermore, Na-deoxycholate is among the bile salts known to create stable gels [62], making it valuable as both an antimicrobial agent and a carrier for topical skin applications, especially in the context of burns and chronic wound infections with MDR *A. baumannii* (i.e., Iraquibacter). Therefore, the findings regarding natural bile salts like Na-deoxycholate and Na-chenodeoxycholate are highly encouraging as potential small molecule biofilm inhibitors. Bile salts could be incorporated into wound dressings or coatings for medical devices, which might enhance their effectiveness in preventing biofilm formation, thereby reducing the risk of infection. Also, it could be applied directly to wounds or surgical sites prone to infection by drug-resistant bacteria. Given their ability to disrupt biofilm formation, they could prevent the establishment or persistence of infections, particularly in chronic wounds where biofilms are common. However, further research is necessary to explore bile salts clinical applications. This includes studying their pharmacokinetics, toxicity, and efficacy in vivo, as well as potential delivery mechanisms for therapeutic use.

## 4. Materials and Methods

### 4.1. Bacterial Strains

Twenty-nine *A. baumannii* strains and two strains belonging *A. calcocaceticus*–*A. baumannii* (Acb) complex were used in this study. Two strains were from the American Type Culture Collection (ATCC 19606 and ATCC BAA747, Rockville, MD, USA), and one from the National Collection of Type Cultures (NCTC 13420, Public Health England, London, UK). The *Pseudomonas aeruginosa* reference strain (ATCC 15692/PAO1) was used as a quality control strain in the lectin production test. *A. baumannii* strains used in this study were previously characterized as MDR, where twenty strains were outpatients and clinical wound isolates [44] and eight environmental strains [63]. The strains were stored in Luria–Bertani broth (LB) supplemented with glycerol (10% *v*/*v*) at −70 °C. The overnight cultures in LB were used for the experiments. All the experiments were conducted using Muller–Hinton agar or broth, unless stated otherwise.

### 4.2. Phenotype Traits

#### 4.2.1. Autoaggregation

The autoaggregation test [64] was used as an indication of the various *A. baumannii* strains cells potential to interconnect and form groups, i.e., autoaglutinates. The optical density was read on the MultiscanGo (ThermoScinetific, Vantaa, Finland) at 650 nm and the degree of autoaggregation was determined. The degree of autoaggregation (AA) was determined using the following equation:

% AA = [(OD_0_ − OD_60_)/OD_0_] × 100, (1)

wherein OD_0_ refers to the optical density of the untreated series, while OD_60_ implies the optical density of the treated series. The test was carried out in three independent repetitions, and the mean values of the obtained results with standard deviation were presented graphically using the GraphPad Prism (version 9.0.0 for Windows, GraphPad Software, Boston, MA, USA, www.graphpad.com). Bacterial isolates in which the AA rate was >50% were considered to be very autoaggregative, moderate autoaggregative isolates had an AA degree of 20–50%, while isolates in which the AA rate was <20% were considered non-autoaggregative [65].

#### 4.2.2. Hydrophobicity

The bacterial adherence to hydrocarbons (BATH) test [66] was used to determine the hydrophobicity of *A. baumannii* isolates. Hydrocarbon n-hexadecane was used. The experiment was conducted in triplicate, and optical density was read on MultiscanGo (ThermoScinetific, Vantaa, Finland) at 560 nm (A_1_). The result values are expressed as the percentage of cells adhering to n-hexadecane (A) relative to the control suspension (A_0_):

A = [(A_0_− A_1_)/A_0_] × 100. (2)

The test was conducted in three independent repetitions, with the mean values of the obtained percentages with standard deviation presented graphically using the GraphPad Prism (version 9.0.0 for Windows, GraphPad Software, Boston, MA, USA, www.graphpad.com). The results were interpreted as follows: strains whose values were >50% were considered to be strongly hydrophobic, moderately hydrophobic when values were in the range of 20–50%, and hydrophilic when the values obtained were <20% [66].

#### 4.2.3. Twitching Motility

For the examination of twitching motility (motility using pilli type IV), *A. baumannii* isolates were used with LBA medium with 1.5% agar [67]. In the case of zones having an irregular shape, three diameters were measured, and the mean value was calculated. This test is performed in three independent repetitions, and the results are represented as the arithmetic mean value of all three replicates with a standard deviation. The isolates were classified as non-motile (NM, <5 mm), intermediately motile (IM, 5–20 mm), and highly motile (HM, >20 mm).

#### 4.2.4. Lectins Production and Semi-Quantification

In order to determine the ability of the lectins production as well as the characterization of the lectins produced by *A. baumannii* isolates, the specificity of bacterial lectins for different blood groups was tested. For this purpose, three blood groups, O Rh+, A Rh+, and B Rh+, were used, and the test was performed using 3% human erythrocyte solution [32]. As a negative control, 0.9% sterile saline was used with 3% human erythrocyte solution in the same ratio. The experiment was made in the same way for each of the examined blood groups, and the presence of lectins is seen as a red precipitate at the bottom of the U-well microtiter plate.

The determination of the lectin production titer was carried out by a modified ex vivo semi-quantitative method using a haemagglutination test [32]. For an ex vivo haemagglutination test, a 3% solution of erythrocyte A Rh+ group was used. In the U-bottom microtiter plates, double dilutions of the supernatant of the centrifuged bacterial suspensions were made, which in the final volume was double diluted from 2 to 2048 times. The sterile saline and the uninoculated substrate were used as negative controls. Also, reference strain *Pseudomonas aeruginosa* PAO1 was used as a positive control. Agglutination of erythrocytes was monitored in the next 30 min to 1 h. The value of the highest dilution of the supernatant that led to haemagglutination and gave a positive reaction was considered to be the lectin titer. Wells of microtiter plates in which hemagglutination occurred with any dilution of the supernatant were recorded as positive for the presence of lectins. The test was carried out in three independent repetitions, and the results obtained were tabulated as the modus value of all three repetitions.

#### 4.2.5. Biofilm Production

The quantification of the formed biofilm by *A. baumannii* wound isolates was carried out by a modified method in microtiter plates, which is used to estimate the potential of bacteria to form a biofilm [68]. The determination of the biofilm formation potential was carried out with *A. baumannii* cultures grown for 24 h. The test was performed in three independent repetitions, and the obtained results were presented graphically as an arithmetic mean with standard deviation using the GraphPad Prism (version 9.0.0 for Windows, GraphPad Software, Boston, MA, USA, www.graphpad.com).

The cell adhesion estimation was performed through the ODc value, which represents the mean value of the optical density of the negative control (OD) for the tested microtiter plate increased for three standard deviations obtained for negative controls. Isolates are classified according to the following criteria:

OD ≤ ODc = non-adherent,

ODc< OD ≤ (2 × ODc) = weakly adherent,

(2 × ODc) < OD ≤ (4 × ODc) = moderately adherent,

(4 × ODc) < OD = strongly adherent [69].

### 4.3. Genotype Traits

The genotyping of *Acinetobacter baumannii* wound isolates was performed to detect genes that are considered important for the virulence of strains and/or for the biofilm-forming potential of the strains. In this test the *bap* gene as well as the gene for PER-1 type β-lactamase were analyzed.

PCR amplification of the fragment of each individual gene was performed in 20 μL of the total volume. The reaction mixture contained 100 ng of each primer, 1 U Green Taq polymerase, 200 μM dNTPs, and 65–550 ng bacterial DNA buffer (Green buffer, Thermoscientific). PCR amplification conditions and primer sequences varied depending on the target gene: *bap* [70] or *bla*_PER-1_ [71] (Table 3). Amplified fragments for the *bla*_PER-1_ gene were detected and analyzed on a 1% (*w*/*v*) agarose gel, while for amplified fragments of *bap* gene detection was performed on a 2% (*w*/*v*) agarose gel. For the visualization and photographing of gels, BioDocAnalyze transilluminator (Biometra, Göttingen, Germany) equipped with a digital camera (Cannon EOS 100D, Tokyo, Japan) was used.

### 4.4. Examination of Bile Salts Effects on MDR A. baumannii

#### 4.4.1. Effect of Bile Salts on Total Growth

The activity of bile salts as alternative antibacterial agents was determined by the broth microdilution susceptibility testing method according to CLSI [72]. The two-fold dilutions of bile salts in sterile distilled water were prepared in the 96-well microtiter plates and mixed with inoculated double-strength Mueller-Hinton broth in a ratio of 1:1 (*v*/*v*). The final bacterial count was approx. 1 × 10^6^ CFU mL^−1^. The final concentrations of tested bile salts were in a range from 32 to 0.25 mg mL^−1^. Microtiter plates were incubated overnight at 37 °C; wells were supplemented with 2,3,5-*triphenyltetrazolium chloride* (TTC) solution to obtain final conc. 0.1% and the microtiter plates were incubated for additional 2 h at 37 °C until red color of formazan appeared in the control wells. The minimum concentration of bile salt required to prevent the appearance of red color, i.e., formation of formazan, was considered a MIC. The experiment was performed in triplicate on two independent occasions and represented as geometrical means of replications. For this test, eight *A. baumannii* isolates (Aba-2572, Aba-4804, Aba-5055, Aba-8781, Aba-8833, Aba-3496, Aba-4010, and ATCC 19606) were selected according to their potential to form a biofilm, as well as the degree of sensitivity to conventional antimicrobial agents. The sensitivity of three *A. baumannii* strains ATCC 19606, Aba-4914, and Aba-5055 to bile salts was previously reported [16].

#### 4.4.2. Effect of Bile Salts on Biofilm Formation

**Figure 4:**
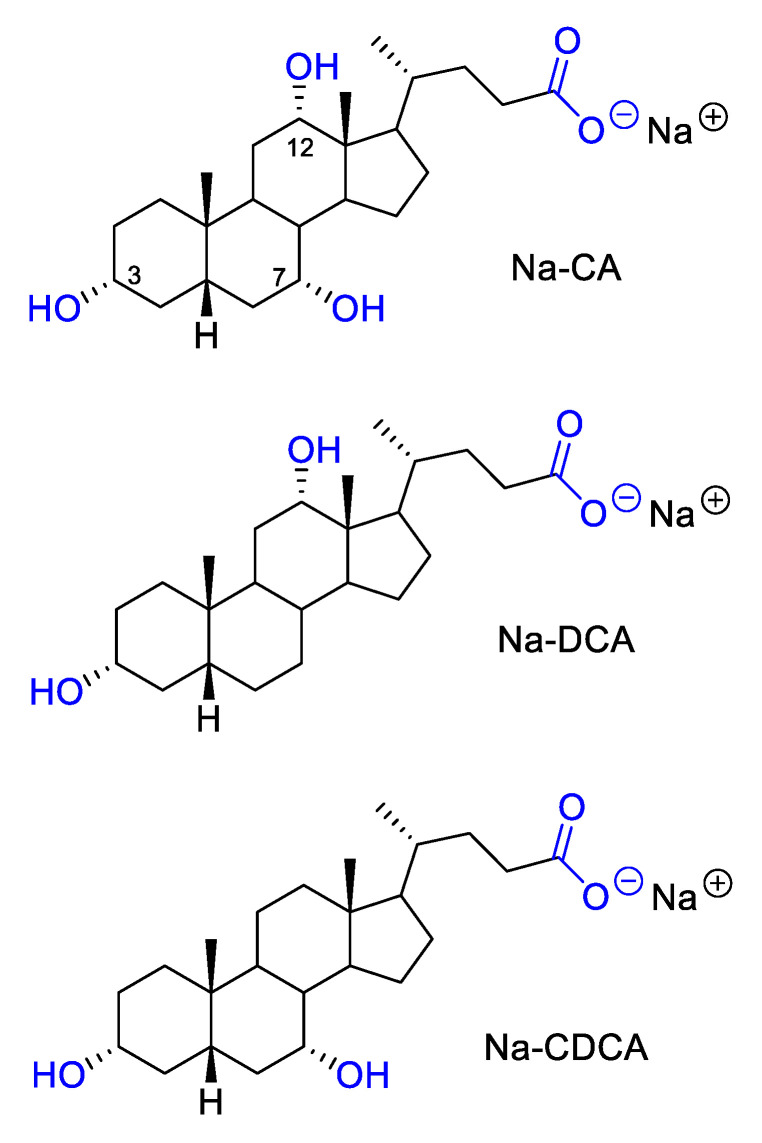
Structure of tested bile salts: Na-CA, Na-DCA and Na-CDCA.

The effect of selected bile salts on the process of biofilm formation was determined by a modified method in microtiter plates, which is used to assess the potential of bacteria to form biofilm [68]. In the test, previously tested solutions of sodium cholate, sodium deoxycholate, and sodium chenodeoxycholate where used for the treatment of bacterial strains (Figure 4) [16].

Two-fold dilutions of bile salt solutions in sterile distilled water were made in a microtiter plate. The final concentrations ranged from 0.25 to 32 mg mL^−1^. For all tested strains, cell suspensions were prepared in PBS buffer and inoculated into a double-concentrated MHB medium. Inoculated medium was added to the wells of a microtiter plate with bile salt dilutions in the ratio 1:1 (*v*/*v*). The final cell count in each well was approx. 1.00 × 10^5^ CFU mL^−1^. Controls were non-inoculated medium and inoculated medium with each strain individually in the same final abundance as in treatments. The microtiter plates were incubated for 24 h at 37 °C.

After the incubation period, the biofilm was quantified according to the method described by Knezevic and Petrovic [68]. Absorbance in microtiter plate wells was measured at 595 nm on a MultiscanGo microtiter plate reader (Thermoscinetific, Vantaa, Finland). The experiment was performed in three independent replicates. Calculations of the biofilm production degree were performed using Microsoft Office Excel^®^ 2007 software (12.0.4518.1014), where the OD values of the produced biofilm degree under the influence of bile salts were first reduced by a negative control and then proportionally compared with a positive control, which represented the maximum value of biofilm production of one strain. The results are expressed as a percentage and presented graphically using GraphPad Prism (version 9.0.0 for Windows, GraphPad Software, Boston, MA, USA, www.graphpad.com).

#### 4.4.3. Effect of Bile Salts on Planktonic Biofilm Cells

Before the quantification of the formed biofilm, quantification of planktonic cells in microtiter plates was performed, in order to determine the effect of bile salts on planktonic cells in the medium during the biofilm formation process. After a 24 h incubation, during which different concentrations of bile salts affected the biofilm formation process and planktonic cells present in the medium during this process, the planktonic cell suspension was transferred to new microtiter plates with MHB and 0.1% TTC solution added to all wells in a ratio of 5:14:1 (*v*/*v*/*v*). The total volume of each well was 200 μL. Microtiter plates were incubated for 3 h at 37 °C, and absorbance was read at 540 nm on a MultiscanGo microtiter plate reader (ThermoScinetific, Vantaa, Finland).

The results of planktonic cell quantification were calculated in the same way as for the effect of bile salts on the biofilm formation process (see Section 4.4.2) and presented graphically using the program GraphPad Prism (version 9.0.0 for Windows, GraphPad Software, Boston, MA, USA, www.graphpad.com).

### 4.5. Statistical Analysis

Correlations between the examined characteristics of the isolates were determined using the non-parametric Kendall tau test, and the differences in characteristics between the isolates originating from wounds and the environment were tested with the Wilcoxon test. Both tests were performed using the Statistica 10 program (StatSoft, Inc., Tulsa, OK, USA, 2011). The obtained correlations and differences were considered statistically significant when *p* ≤ 0.05.

## 5. Conclusions

The findings presented in this study underscore the pressing need for effective solutions in combating antibiotic resistance and biofilm production in *A. baumannii*. While differences in biofilm production were observed between environmental and wound isolates, resistance and biofilm-associated traits were shared among strains from both origins. None of the examined features directly correlate with the observed high biofilm production, confirming that biofilm formation is a multi-factorial process with no single dominant cell trait influencing its development. Instead, various traits contribute together to enhance this remarkable bacterial ability, making it extremely difficult to eradicate.

The promising results obtained with the natural bile salts Na-chenodeoxycholate and Na-chenodeoxycholate as potential small-molecule biofilm inhibitors highlight their potential as a valuable tool in the fight against biofilm-related infections, shedding light on a novel avenue for future research and potential therapeutic applications. Moreover, additional modifications of bile salts and/or their conjugation with other antimicrobials offer opportunities for further research, aiming to enhance their efficacy and broaden their spectrum of activity against a wider range of pathogens. These directions warrant further research to develop innovative strategies for addressing the challenge of antibiotic resistance and improving patient outcomes, especially in clinical settings.

## Figures and Tables

**Table 1:** Twitching motility, titre of the lectins, lectins specificity to different blood types and presence of the *bap* and *bla*_PER-1_ genes in genomes of MDR Acb isolates.

Origin of the Isolates	Isolate Designation	Diffuse Blur Zone Diameter (mm)	Classification ^1^	Titre of the Lectins	Blood Types ^2^			Genes	
					O Rh+	A Rh+	B Rh+	*bap*	*bla*_PER-1_
Reference strains	ATCC BAA747	22.0 ± 1.8	HM	1/16	+	+	+	+	−
	ATCC 19606	20.5 ± 1.3	HM	1/4	+	+	+	+	−
	NCTC 13423	7.5 ± 1.3	IM	1/16	+	+	+	+	−
	PAO1	26.0 ± 1.8	HM	1/32	+	+	+	/	/
Human pathological material isolates	Aba-2572	18.0 ± 5.2	IM	1/4	+	+	+	+	−
	Aba-2793	25.5 ± 5.3	HM	1/16	+	+	+	+	−
	Aba-4156	33.0 ± 7.2	HM	1/16	+	+	+	+	−
	Aba-4727	20.5 ± 2.0	HM	1/32	+	+	+	+	+
	Aba-4779	16.0 ± 1.8	IM	1/32	+	+	+	+	+
	Aba-4803	51.5 ± 6.0	HM	1/32	+	+	+	+	−
	Aba-4804	21.5 ± 5.7	HM	1/64	+	+	+	+	−
	Aba-4890	23.8 ± 4.6	HM	1/64	+	+	+	+	−
	Aba-4914	9.5 ± 1.3	IM	1/16	+	+	+	+	−
	Aba-5055	14.5 ± 0.6	IM	1/32	+	+	+	+	−
	Aba-5074	11.5 ± 1.3	IM	1/32	+	+	+	+	+
	Aba-5081	19.3 ± 0.3	IM	1/16	+	+	+	+	+
	Aba-5372	22.5 ± 2.1	HM	1/8	+	+	+	+	+
	Aba-6673	27.8 ± 1.2	HM	1/16	+	+	+	+	+
	Aba-7860	34.0 ± 5.9	HM	1/32	+	+	+	+	+
	Aba-8255	21.0 ± 7.0	HM	1/16	+	+	+	+	+
	Aba-8781	46.0 ± 1.63	HM	1/4	+	+	+	+	−
	Aba-8833	26.8 ± 1.2	HM	1/4	+	+	+	+	−
	Aba-3496	26.5 ± 1.3	HM	1/4	+	+	+	+	−
	Aba-4010	16.5 ± 3.9	IM	1/8	+	+	+	+	−
Environmental isolates	Aba-S-Ace	12.0 ± 1.6	IM	1/16	+	+	+	+	−
	Aba-S-Tyr	19.0 ± 4.2	IM	1/32	+	+	+	+	−
	Aba-DZ-Ace	15.0 ± 1.8	IM	1/4	+	+	+	+	−
	Aba-DN-Ace	52.5 ± 11.1	HM	1/8	+	+	+	+	+
	Aba-M-Ace	14.5 ± 1.3	IM	1/16	+	+	+	+	−
	Aba-DP-Phe	19.8 ± 4.4	IM	1/4	+	+	+	+	−
	Aba-DTD-Tyr	28.8 ± 3.9	HM	1/32	+	+	+	+	−
	Aba-B-Phe	17.0 ± 1.8	IM	1/32	+	+	+	+	−

^1^ NM (non-motile, <5 mm), IM (intermediately motile, 5–20 mm), HM (highly motile,>20 mm); ^2^ (+) the presence, (−) the absence, (/) non tested.

**Table 2:** Sensitivity of MDR *A. baumannii* strains to bile salts (mg mL^−1^).

*A. baumannii* Strains	Bile Salts MIC ^1^			Bile Salts BIC_50_^2^			Bile Salts BIC_90_^3^		
	Na-CA ^4^	Na-DCA ^5^	Na-CDCA ^6^	Na-CA	Na-DCA	Na-CDCA	Na-CA	Na-DCA	Na-CDCA
ATCC 19606 ^8^	10.1	0.8	1.0	1.0	1.0	4.0	32.0	2.0	8.0
Aba-2572	0.7	0.25	0.5	4.0	0.5	1.0	32.0	8.0	8.0
Aba-4804	8.0	0.35	0.5	16.0	1.0	1.0	NA	2.0	4.0
Aba-4914 ^8^	8.0	0.5	1.4	NA	2.0	1.0	NA	8.0	4.0
Aba-5055 ^8^	8.0	0.7	1.0	32.0	2.0	2.0	32.0	8.0	4.0
Aba-8781	5.7	0.5	2.0	32.0	2.0	4.0	NA ^7^	8.0	8.0
Aba-8833	0.7	0.25	0.35	1.0	0.25	0.25	16.0	1.0	1.0
Aba-3496	0.7	0.25	0.35	4.0	0.25	1.0	16.0	1.0	4.0
Aba-4010	4.0	0.35	0.7	8.0	1.0	1.0	16.0	2.0	2.0

^1^ MIC—Minimal Inhibitory Concentration, ^2^ BIC_50_—Biofilm Inhibitory Concentration required to inhibit biofilm formation for ≥50%, ^3^ BIC_90_—Biofilm Inhibitory Concentration required to inhibit biofilm formation for ≥90%, ^4^ Na-CA—sodium-cholate, ^5^ Na-DCA—sodium-deoxycholate, ^6^ Na-CDCA—sodium-chenodeoxycholate, ^7^ NA—not active, ^8^ MIC values were previously reported in Aleksic Sabo et al. [16].

**Table 3:** Oligonucleotide sequences for PCR analysis of *A. baumannii* isolates.

Primer	Nucleotide Sequence (5′ –> 3′)	PCR Product Size (bp)	Reference
Aba_*bap*-F	TGCTGACAGTGACGTAGAACCACA	823	[31]
Aba_*bap*-R	TGCAACTAGTGGAATAGCAGCCCA		
Aba_*bla*_PER-1_-F	ATGAATGTCATTATAAAAGC	925	[32]
Aba_*bla*_PER-1_-R	AATTTGGGCTTAGGGCAGAA		

## Data Availability

The raw data supporting the conclusions of this article will be made available by the authors on request.
